# GODoc: high-throughput protein function prediction using novel *k*-nearest-neighbor and voting algorithms

**DOI:** 10.1186/s12859-020-03556-9

**Published:** 2020-11-18

**Authors:** Yi-Wei Liu, Tz-Wei Hsu, Che-Yu Chang, Wen-Hung Liao, Jia-Ming Chang

**Affiliations:** Department of Computer Science, National Chengchi University, 11605 Taipei, Taiwan

**Keywords:** Protein function prediction, Machine learning, Gene ontology, Homology extension, Data science

## Abstract

**Background:**

Biological data has grown explosively with the advance of next-generation sequencing. However, annotating protein function with wet lab experiments is time-consuming. Fortunately, computational function prediction can help wet labs formulate biological hypotheses and prioritize experiments. Gene Ontology (GO) is a framework for unifying the representation of protein function in a hierarchical tree composed of GO terms.

**Results:**

We propose GODoc, a general protein GO prediction framework based on sequence information which combines feature engineering, feature reduction, and a novel ​*k*​-nearest-neighbor algorithm to resolve the multiple GO prediction problem. Comprehensive evaluation on CAFA2 shows that GODoc performs better than two baseline models. In the CAFA3 competition (68 teams), GODoc ranks 10th in Cellular Component Ontology. Regarding the species-specific task, the proposed method ranks 10th and 8th in the eukaryotic Cellular Component Ontology and the prokaryotic Molecular Function Ontology, respectively. In the term-centric task, GODoc performs third and is tied for first for the biofilm formation of *Pseudomonas aeruginosa* and the long-term memory of *Drosophila melanogaster*, respectively.

**Conclusions:**

We have developed a novel and effective strategy to incorporate a training procedure into the *k*-nearest neighbor algorithm (instance-based learning) which is capable of solving the Gene Ontology multiple-label prediction problem, which is especially notable given the thousands of Gene Ontology terms.

## Background

Proteins are important macromolecules in living organisms because they carry essential functions to ensure the survival of creatures. If we know what function a protein carries, we can understand life at the molecular level and the molecular mechanisms of disease. Gene Ontology (GO) is the main framework for unifying the representation of protein function, initiated by the GO Consortium in 1998. GO classifies functions into three domains: Biological Process Ontology (BPO), Cellular Component Ontology (CCO), and Molecular Function Ontology (MFO). BPO describes the biological process in which the gene product participates, CCO specifies the location of the gene product, and MFO indicates what the gene product can do or its ability. GO terms are linked to each other with a hierarchical directed tree structure. The relationship and terms can be graphed as directed edges and nodes, respectively (an example can be found on page 26, [1]). GO is annotated to a protein by either biological experiments or computational prediction. Therefore, each GO annotation is associated with an evidence code to indicate the method employed to generate the annotation [2].

Compared with the growth of protein sequence data, the speed of protein function annotation from wet lab experiments is slow. Fortunately, computational function prediction can help wet labs formulate biological hypotheses and prioritize experiments. In this research, we seek to use information about a protein sequence to predict its GOs, a multiple-label classification problem. This task is different from traditional multiple-label classification, as GO labels are hierarchical. There exist both computational and biological challenges. Computational speaking, the number of annotated proteins is relatively small compared with the number of GO terms. There are about 40,000 unique GO terms, but only 66,841 experimentally annotated sequences in Swiss-Prot, as of September 2016. Biologically, annotations might not be perfectly reproduced due to budgetary or ethical reasons. In addition, some experiments are performed in vitro and may not reflect a protein’s activity in vivo.

Predicting the function of a target protein from its homologs is the most common approach. Homologs between two proteins may indicate a common ancestry; thus they may have the same function. As a result, the available GOs of the homologs are prediction candidates for a target protein. The Basic Local Alignment Search Tool (BLAST) and Position-Specific Iterated BLAST (PSI-BLAST) [3] are two standard tools for searching homologous sequences. PSLDoc uses the information from PSI-BLAST, the position-specific scoring matrix (PSSM), to build a TFPSSM (Term Frequency based on PSSM) feature to find homologous proteins [4], and PSLDoc is used to predict protein subcellular localization, which can be considered as a subset of CCO. In addition to sequence-similarity-based approach, there exist some databases or computational methods to characterize proteins to individual domains or motifs, which are useful for function prediction. For example, CATH-Gene3D clusters proteins into functional families (FunFam), which implies similar sequences, structures, and GOs [5]. The CATH FunFHMMer web server identifies FunFams for an unknown target such that FunFam associated GOs are good prediction candidates [6].

The Critical Assessment of Functional Annotation (CAFA) aims to evaluate prediction methods in an unbiased way. It was established by the Function Special Interest Group (Function-SIG). CAFA1, CAFA2, CAFA3 and CAFA *π* (3.14), the first four challenges, were organized and carried out during 2010–2011 [7], 2013–2014 [8], 2016–2017 [9], and 2017–2018 [9], respectively. The competitions are conducted in a time-delayed format with a *prediction* phase, an *annotation growth* phase, and an *evaluation* phase (Fig. 12, [9]). At the beginning, the organization provides a large number of protein sequences with unknown function for the participants to predict (*t*_− 1_ in Fig. 12, [9]). During the *prediction* phase, the predictor submits their predicted annotations of these target proteins. When the *prediction* phase ends (*t*_0_ in Fig. 12, [9]), the challenge moves on to the *annotation growth* phase (*t*_1_ in Fig. 12, [9]), in which some protein function of target proteins might be annotated through experiment. Entering the *evaluation* phase, those proteins with annotations are selected as a benchmark to evaluate each method’s prediction performance.

We propose GODoc, an effective GO prediction framework [10] extended from PSLDoc [4] and PSLDoc2 [11], which has demonstrated excellent performance in predicting protein subcellular localization. We design three novel voting strategies based on the *k-nearest-neighbor* algorithm by incorporating a training procedure to solve the multiple-label prediction problem, as the number of GO terms is much larger than the number of localization sites (Section: Data sets). GODoc is evaluated on the CAFA2 and CAFA3 data sets and yields significantly better results than the two baseline models. In the CAFA3 competition, GODoc ranks 3rd and 5th among 67 and 59 methods in full and partial modes, respectively. According to the minimum normalized semantic distance metric in BPO, and for CCO, it achieves a score of 0.592, which ranks among the top 10% in 67 methods based on Fmax.

## Results

### Experiment 1: PCA

**Fig. 1:**
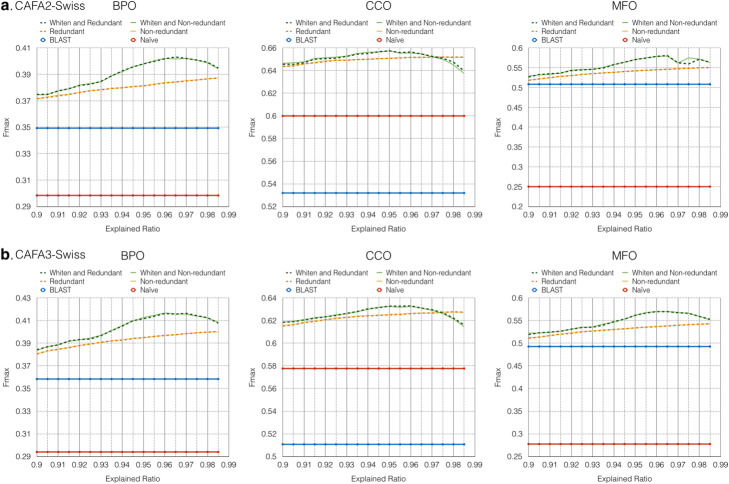
Fmax of TFPSSM 1NN on CAFA2-Swiss (**a**) and CAFA3-Swiss (**b**) with different PCA parameters, where the explained ratio ranges from 90 to 98.5% with a step size of 0.5%

The Fmax of two baseline models and TFPSSM 1NN with different PCA parameters are summarized in Fig. 1. TFPSSM 1NN demonstrates better performance than the two baseline models on both the CAFA2-Swiss (Fig. 1a) and CAFA3-Swiss (Fig. 1b) datasets. TFPSSM extracts homology information from BLAST search, which has been shown to be efficient against a non-redundant database without losing prediction performance [11]. This has also been confirmed by our experiment because the dashed (original) and solid (non-redundant) lines of the same color are almost identical (compatible Fmax), which shows that the TFPSSM 1NN algorithm effectively picks out neighbors by keeping one representative of redundant sequences (non-redundant dataset). During feature reduction, whitening pre-processing (green versus yellow/orange) yields the best explained ratios of around 96%. These parameter settings are used in further experiments.

### Experiment 2: *k*-nearest-neighbor algorithm and weighted voting

**Fig. 2:**
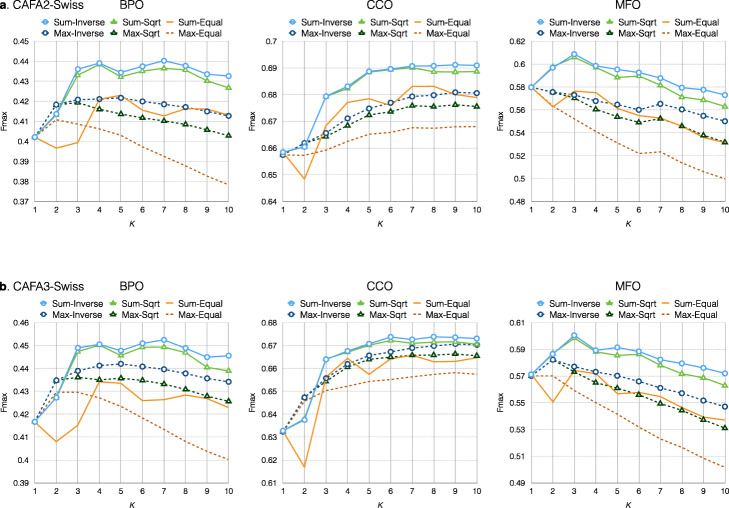
Fmax of Fixed-KNN on CAFA2-Swiss (**a**) and CAFA3-Swiss (**b**) with different *k*, voting schemes, and propagations, in which Sum-* and Max-* denote the Sum and Max propagation function, respectively

In the Fixed-KNN experiment, we set *k* from 1 to 10 and voted with Sum and Max propagation functions using three different weight assignment rules (Fig. 2). We observe that better performance can be obtained by setting *k* larger than 1 in BPO and CCO. However, the benefit decreases for *k* larger than 3 in MFO. Among the three weight schemes, *Inverse* is more reliable than the other two methods. As a result, we employ the *Inverse* approach in further experiments. The results also reveal that Sum propagation is better than Max.

**Fig. 3:**
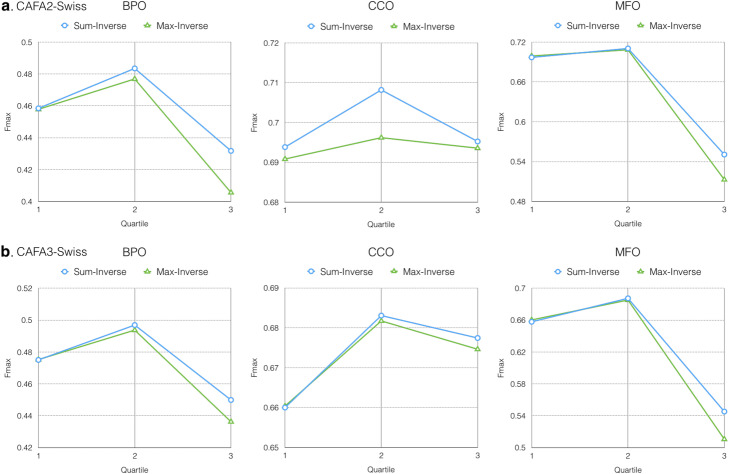
Fmax of Dynamic-KNN on CAFA2-Swiss (**a**) and CAFA3-Swiss (**b**) with different dynamic threshold and propagation functions under partial evaluation mode

**Fig. 4:**
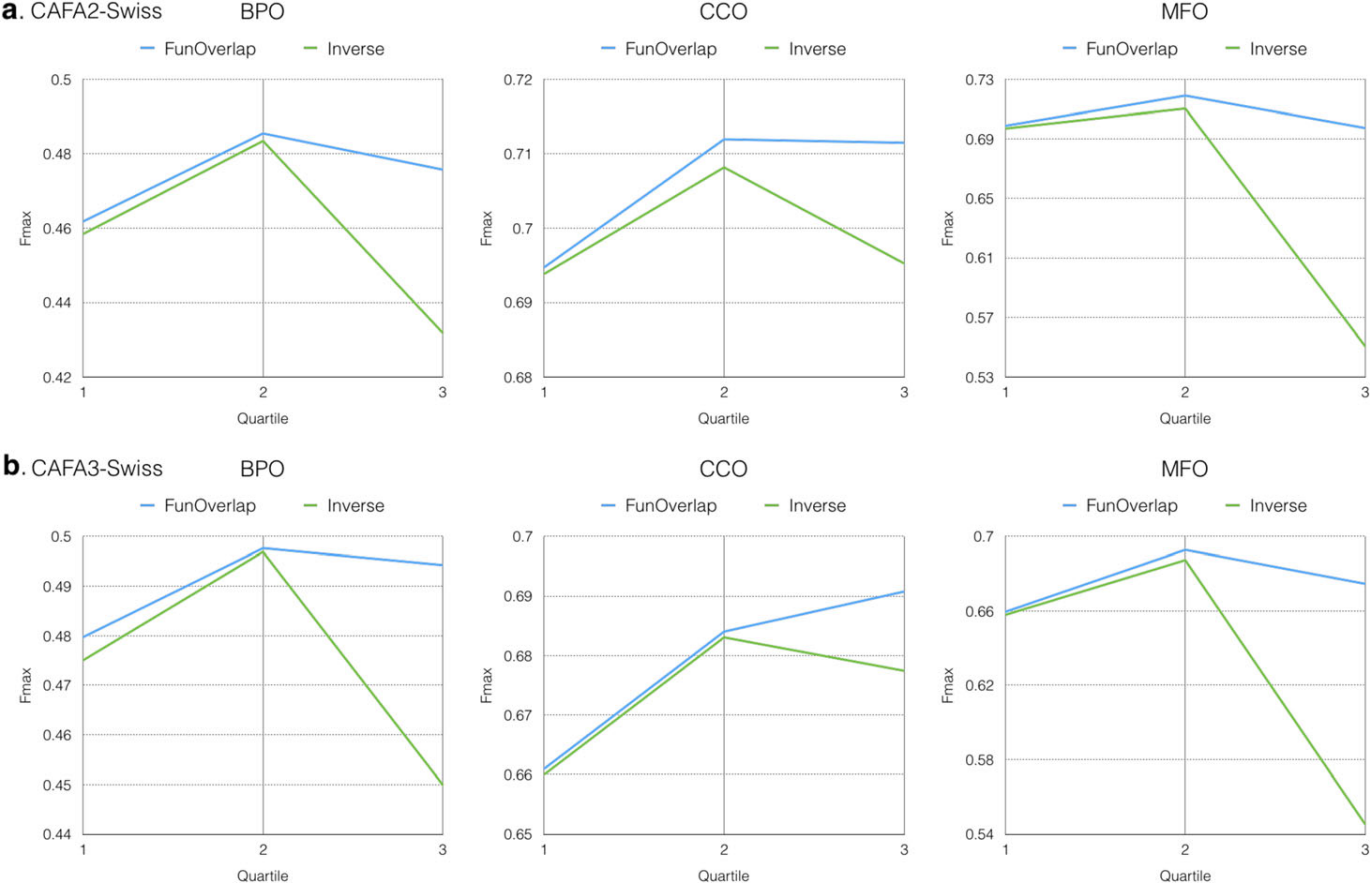
Fmax of Dynamic-KNN on CAFA2-Swiss (**a**) and CAFA3-Swiss (**b**) with different dynamic threshold and voting weight schemes under partial evaluation mode

Figure 3 presents Fmax of Dynamic-KNN under different distance thresholds and propagation functions. Using the 2nd quartile (Q2) as the threshold not only yields the best performance on the three ontologies, but also contains half of the test data (Q2, *Inverse* in Table 1). In this experiment, we also find the Sum-propagated function to be an effective way to address our problem. Given the previous two experiments (Figs. 2 and 3), we conclude that *Sum* propagation is better than *Max*; hence in the following experiments, to reduce training complexity, we consider only *Sum* propagation. The comparison between the *Inverse* and *FunOverlap* voting weight schemes is shown in Fig. 4. Although FunOverlap outperforms Inverse, it predicts fewer proteins (# of preds and % in Table 1).

**Table 1:** Dynamic-KNN coverage in partial model with respect to different distance thresholds and voting weight schemes in the cross-validation validation data set. *# of seqs*: total number of proteins in the set. *Distance*: distance threshold used in Dynamic-KNN. *# of preds*: number of predicted proteins and its corresponding proportion in %

Type	Dataset	# of seqs	Distance	*Inverse*		* FunOverlap*	
				# of preds	%	# of preds	%
BPO	CAFA2-Swiss	8146	Q1	2046	25.12	1882	23.10
			Q2	4095	50.27	3654	44.86
			Q3	6112	75.03	5167	64.43
	CAFA3-Swiss	10,163	Q1	2562	25.21	2309	22.72
			Q2	5095	50.13	4470	43.98
			Q3	7601	74.79	6333	62.32
CCO	CAFA2-Swiss	8114	Q1	2039	25.13	1855	22.86
			Q2	4034	49.72	3540	43.63
			Q3	6042	74.46	4898	60.36
	CAFA3-Swiss	9866	Q1	2548	24.91	2204	22.34
			Q2	4922	49.89	4261	43.19
			Q3	7357	74.57	5912	59.92
MFO	CAFA2-Swiss	5211	Q1	1291	24.77	1204	23.11
			Q2	2593	49.76	2366	45.40
			Q3	3902	74.88	3405	65.35
	CAFA3-Swiss	7017	Q1	1756	25.02	1630	23.23
			Q2	3518	50.14	3185	45.39
			Q3	5278	75.22	4573	65.17

**Fig. 5:**
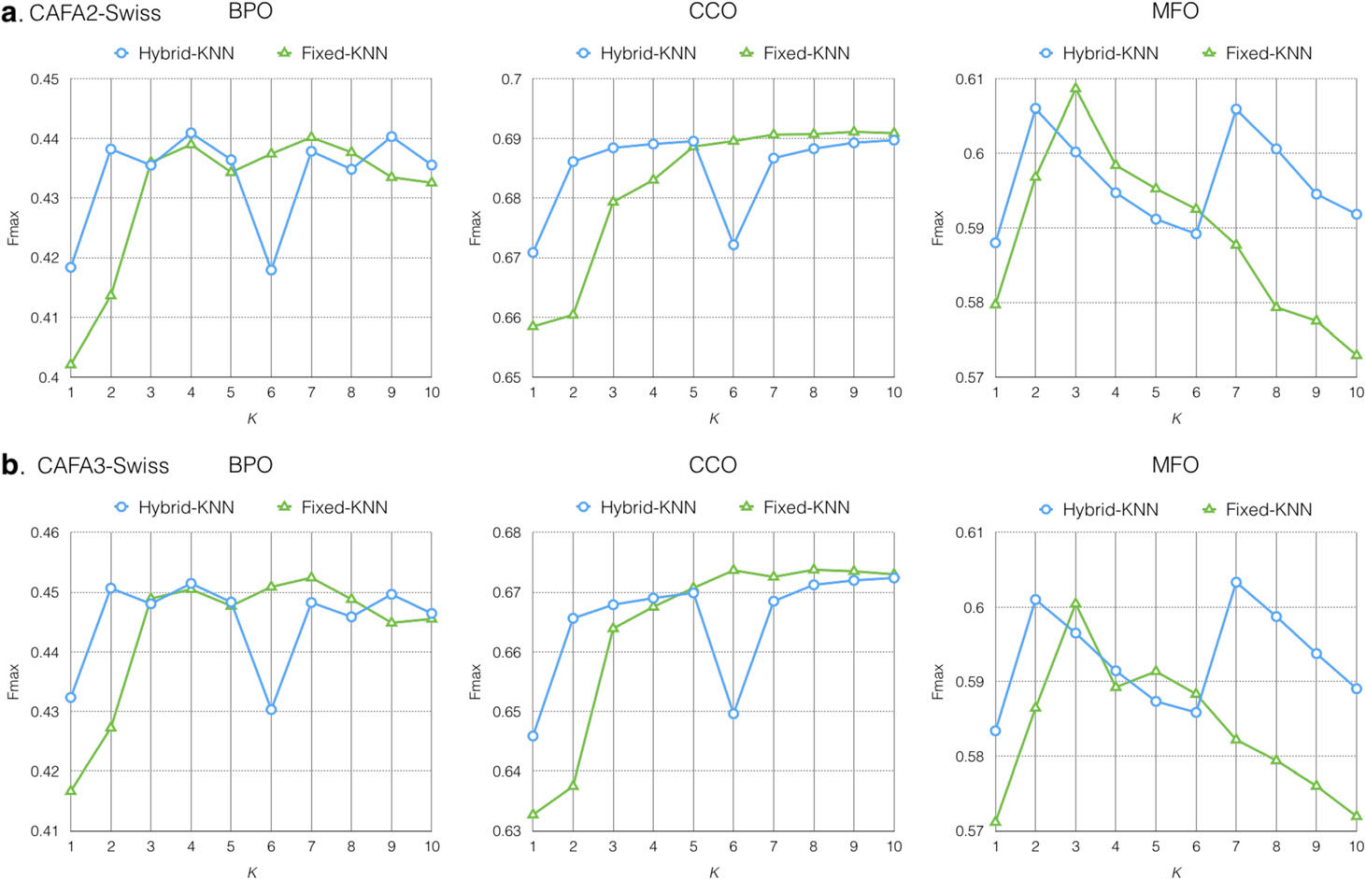
Fmax of Hybrid-KNN and Fixed-kNN on CAFA2-Swiss (**a**) and CAFA3-Swiss (**b**)

In the Hybrid-KNN experiment, we seek to study the effects of combining Fixed-KNN and Dynamic-KNN. We examine the combination of fixed *k* from 1 to 10 and the 2nd quartile as a dynamic threshold with *Inverse* voting weight and *Sum* propagation (Fig. 5). We observe no clear benefit in Fmax from the combination of Fixed-KNN and Dynamic-KNN.

### Summary of experimental results

As determined in the previous experiments, the optimal parameter combination used for CAFA2-Swiss is listed in Table 2. All TFPSSM features are extracted using PCA with *whitening* preprocessing, SVD from the *non-redundant* training dataset, and *Sum* propagation.

**Table 2:** The best parameter combination trained from CAFA2-Swiss is utilized in the CAFA2-Benchmark

Type	PCA dims	Exp. ratio (%)	Method	Voting weight	*k* or *dynamic*
BPO	107	96.0	1NN	–	1
			Fixed-KNN	inverse	7
			Dynamic-KNN	inverse	Q2
			Hybrid-KNN	inverse	4 + Q2
CCO	51	95.0	1NN	–	1
			Fixed-KNN	inverse	9
			Dynamic-KNN	inverse	Q2
			Hybrid-KNN	inverse	7 + Q2
MFO	121	96.5	1NN	–	1
			Fixed-KNN	inverse	3
			Dynamic-KNN	inverse	Q2
			Hybrid-KNN	inverse	3 + Q2

**Fig. 6:**
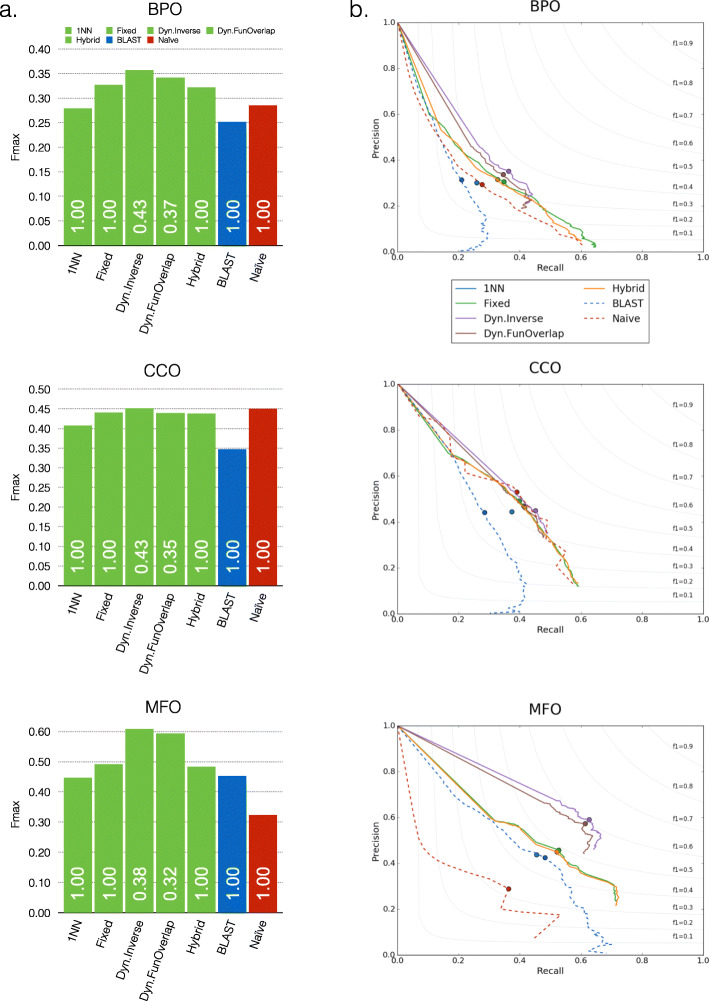
Fmax (**a**) and precision-recall curve (**b**) of each method on CAFA2-benchmark trained on CAFA2-Swiss, where Fixed, Dyn.Inverse, Dyn.FunOverlap, and Hybrid represent Fixed-KNN, Dynamic-KNN with Inverse voting weight, Dynamic-KNN with FunOverlap voting weight, and Hybrid-KNN, respectively. The number inside the bar shows the predicted proportion, in particular for Dynamic-KNN in partial mode

Figure 6 shows the Fmax (a) and precision-recall curve (b) of 1NN, Fixed-KNN, and Hybrid-KNN under full evaluation mode and Dynamic-KNN under partial evaluation mode. Although Dynamic-KNN with *Inverse* weighting performs best among the three Ontologies, it only predicts less than half of the proteins (0.38–0.43). For the full mode, Fixed-KNN and Hybird-KNN show compatible performance, which is better than 1NN. Most of the proposed *k*-nearest-neighbor voting algorithms (green bars) perform better than the two baseline methods, especially in BPO and MFO. The exceptional performance of the Naive method in CCO is biased because the benchmark proteins were annotated with more general terms than the (training) proteins previously deposited in the UniProt database [8].

## Discussion

We participated in the CAFA3 competition as the NCCUCS team. The manuscript summarizing our CAFA3 results was published in Genome Biology and bioRxiv [9, 12]. We submitted three models: 1NN, Fynamic-KNN with Inverse, and Dynamic-KNN with FunOverlap. According to the official evaluation, our methods performed quite well among the 68 teams (Fig. 12 in [12]). In the protein-centric evaluation, it ranks 3rd and 5th in full and partial modes, respectively, according to the minimum normalized semantic distance metric in the BPO ontology (Supplemental Table S1). For the CCO ontology, GODoc (NCCUCS) ranks 10th based on the Fmax metric (Fig. 3c in [9]) and the precision-recall curve (Fig. 3F in [9]). Since different methods sometimes perform differently on different species [9], the benchmarks are further divided into eukaryotic- and prokaryotic-species categories. GODoc ranks 10th and 8th in eukaryotic CCO and prokaryotic MFO Fmax, respectively (Fig. 5c, d in [9]). In addition to the protein-centric task, predicting which proteins are associated with a given function (term-centric, binary classification) is also evaluated in CAFA3. For biofilm formation (GO:0042710) of the bacterium *Pseudomonas aeruginosa*, the proposed method ranks third in AUC (Fig. 9b in [9]). For long-term memory (GO:0007616) of *Drosophila melanogaster*, our method ranks first (tied with other two methods) in AUC (Fig. 10 in [9]).

## Conclusions

We propose a framework for protein function prediction that utilizes TFPSSM features. We propose three different methods, namely, TFPSSM 1NN, TFPSSM Vote (Fixed-KNN, Dynamic-KNN, and Hybrid-KNN), and TFPSSM CATH (Dynamic-KNN with FunOverlap) to enhance prediction accuracy. The advantage of traditional KNN lies in its interpretability. Its performance, however, is inferior to other machine learning methods since no actual training takes place. Here we demonstrate that variants of KNN with extra training procedures (dynamic + voting scheme) can outperform baseline methods.

As a newly developed framework, there are still a wide range of ideas worth investigating in the future (i.e., combining hydrophobe with TFPSSM). In addition, as we have demonstrated the ability of the proposed framework to predict protein subcellular localization and protein function, we expect it to perform effectively on other protein prediction problems as well.

## Methods

Firstly, each protein is represented by TFPSSM, a feature vector based on the frequency of the gapped-dipeptides [4] in the position-specific scoring matrix (PSSM). Then, principal component analysis (PCA) is employed to reduce the TFPSSM features to a lower number of dimensions. Finally, we combine variance *k*-nearest-neighbor algorithms with CATH FunFam information to predict GOs. The details of feature extraction, dimensionality reduction, CATH information, and the voting scheme are described in the following sections.

### Feature representation by TFPSSM

**Fig. 7:**
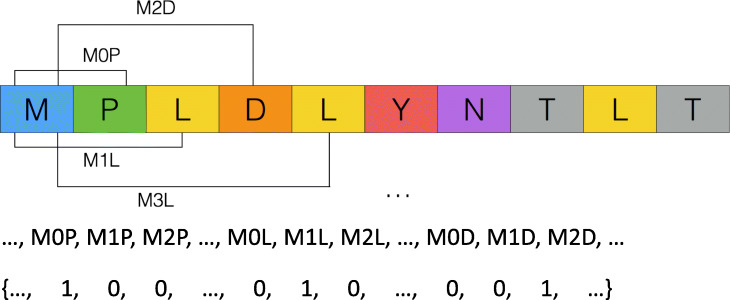
An example of amino acid-coupling pattern representation. Given the protein sequence “MPLDLYNTLT”, it contains amino acid-coupling patterns M0P, M1L, M2D, M3L, and so on. Its corresponding amino acid-coupling pattern is shown in the bottom part

When considering proteins as documents, *n*-peptide is a general term representation [13]: a peptide of length *n* without gaps (bi-gram for *n* = 2). However, as using *n*-peptides to capture long-distance amino acid information results in a high-dimensional vector, the new protein representation *gapped amino acid pair* was proposed [14], later followed by *amino acid-coupling patterns* [15]. In PSLDoc, Chang et al. modify amino acid-coupling patterns to *gapped-dipeptide* [4], in which *XdY* denotes the amino acid coupling pattern of amino acid types *X* and *Y* separated by *d* amino acids (Fig. 7). The vector size is controlled by *l*, where *XdY* for 0 ≤ *d* ≤ *l*. The dimension of gapped-dipeptide is 20 × (*l* + 1) × 20. Taking *l* = 13 as an example, a protein is represented by a gapped-dipeptide feature vector of 5600 (=20 × 14 × 20) dimensions [4].

The PSSM is evolutionary information generated by PSI-BLAST. For a protein sequence *S* of length *n*, the PSSM of *S* is represented by an *n* × 20 matrix, in which the *n* rows correspond to the amino acid sequence of *S* and the columns correspond to the 20 distinct amino acids. In predicting protein localization, Chang et al. propose TFPSSM to combine gapped-dipeptide representation with the PSSM [4]. That is, the frequency of the gapped di-peptides is calculated based on the PSSM. The fast (insensitive) PSI-BLAST parameter setting is used to reduce running times (−matrix BLOSUM80 –evalue 1e-5 –gapopen 9 –gapextend 2 –threshold 999 –seq yes –soft_masking true –numter_iteration 2) [11].

### Dimensionality reduction by principal component analysis

PCA is a useful statistical procedure to decompose high dimension datasets into low dimension ones with a set of successive orthogonal components that explain the maximum variance of the data. Therefore, we use PCA to reduce TFPSSM’s feature dimension.

In our study, the orthogonal components are computed from the training data and applied on both training data and test data using the *Scikit-learn* v0.19.0 module. The size of the reduced dimensions can be chosen to reflect different explained variance ratios. Accordingly, we conducted a series of experiments to determine the appropriate variance ratios based on five-fold cross-validation of CAFA2-Swiss and CAFA3-Swiss (detailed in the Evaluation section, Experimental design, Experiment1). Finally, the distance between two proteins is defined as the Euclidean distance of the two corresponding vectors in PCA projected space.

### Novel *k*-nearest-neighbor algorithms

We propose three strategies to apply the *k*-nearest-neighbor (KNN) algorithm to select candidate proteins. The predicted GOs of the target are determined by voting for results from GOs of the candidate proteins. We use a weighted voting strategy, that is, the greater the similarity to the target, the higher the voting weight. The voting result represents the likelihood of the predicted GOs, which are summarized as *confidence scores*.

**Fig. 8:**
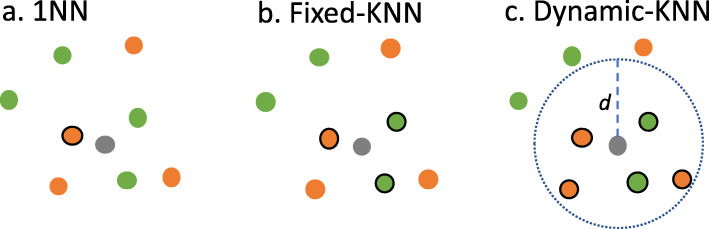
An illustration showing 1NN, Fixed-KNN, and Dynamic-KNN. A query protein is colored in gray. There are two GOs for training data (colored in green and orange) where the neighbor proteins picked are indicated with a solid circle. **a** 1NN: the nearest training protein is selected. **b** Fixed-KNN: three training proteins are picked for *K* = 3. **c** Dynamic-KNN: five proteins are selected, as their distances to the query are smaller than the threshold *d*

1. TFPSSM 1NN: The GOs of the query protein are predicted as the same GOs of its nearest neighbors with a confidence score of 1.00 (Fig. 8a).
2. TFPSSM vote: We propose three ways to choose *k* instead of 1NN.
  - Fixed-KNN: *k* is fixed and chosen based on training data. Figure 8b depicts an example in which *k* is set to 3. *k* is determined based on five-fold cross-validation of CAFA2-Swiss and CAFA3-Swiss (detailed in the Evaluation section, Experimental design, Experiment2).
  - Dynamic-KNN: We calculate the distance distribution of each protein’s nearest neighbors such that we are able to use the 1st, 2nd, or 3rd quartile (Q1, Q2 or Q3) as a distance threshold to select neighbors instead of a fixed *k*, that is, training proteins are selected as neighbors when their distances to the query protein are smaller than the threshold. Figure 8.c shows an example for the threshold as *d*. Dynamic-KNN is not applicable to those query proteins when no neighbor is closer than the given distance threshold. The evaluation of Dynamic-KNN is done following the process of the partial model in CAFA3, involving only benchmarking predicted queries.
  - Hybrid-KNN: if a protein cannot be predicted by Dynamic-KNN, we apply fixed-KNN to select the *k*-nearest neighbors ignoring the distance threshold. This is considered the combined prediction of Fixed-KNN and Dynamic-KNN.

### Voting weight schemes

When neighbor proteins are selected, their voting weights are determined as follows: (1) *Equal*: all weights are equal to 1; (2) *Inverse*: inverse of the Euclidean distance, *d*, between the query protein and the neighbor protein, 1/*d*; (3) *Sqrt-Inverse*: square root of the above inverse weight, $\sqrt{1/d}$, which aims to constrain the range of voting weights to prevent extremely large weights; (4) *FunOverlap*: In addition to the above voting schemes concerning sequence feature space, *FunOverlap* is incorporated to integrate information from protein domains. The domain-based approach has been shown to be useful in predicting protein function [16], where proteins are classified into FunFam. We adopt the HMMer model of FunFams released on the CATH-Gene3D server to predict the FunFams of the query and neighbor proteins with an *E*-value threshold of 10^−5^ [17]. Then, the voting weight of the neighboring protein is set to the overlap proportion between its predicted FunFams and those of the query. The *FunOverlap* item is applicable if there is no FunFam below the *E*-value threshold (10^− 5^) or when the overlap proportion is zero Therefore, *FunOverlap* is only used in Dynamic-KNN.

### GO propagate step

**Fig. 9:**
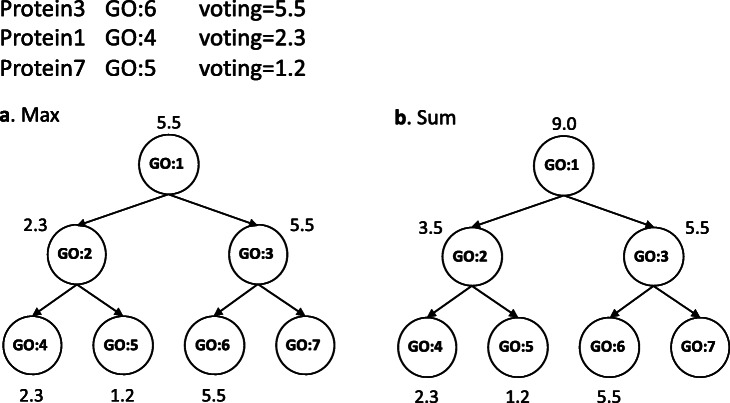
An illustration showing two functions for merging propagated voting weights from child nodes. Given three proteins selected as neighbors by 3-NN, their corresponding GOs and voting weights are shown in the top. **a** *Max*: The voting weight of the parent is the maximum weight of the child nodes. For example, the weight of GO:2 is 2.3, the maximum of 2.3 and 1.2. **b** *Sum*: The voting weight of the parent is the sum weight of the child nodes. For example, the weight of GO:2 is 3.5, the sum of 2.3 and 1.2

Because GO is a hierarchical structure, GO prediction of the node is propagated to its parent node. There are two approaches to merge propagated voting weights from child nodes: *Max* and *Sum* (Fig. 9). The former uses the maximum weight of the child nodes as a weight (Fig. 9a), and the latter uses the sum of the children weights as a weight (Fig. 9b). After the propagation step is finished, the score of each candidate GO is normalized between 0 and 1 by dividing it by the maximum score.

**Fig. 10:**
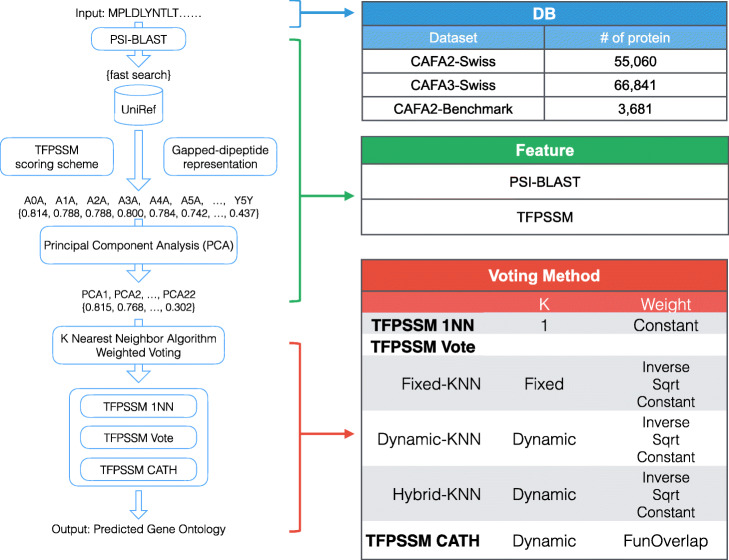
System architecture of GODoc for protein GO prediction, which contains three parts: PSSM homologous extension (blue), TFPSSM feature representation (green), and the proposed voting algorithms (red). The former two parts are based on PSLDoc2 with updated databases. The last part combines a novel *k*-nearest-neighbor algorithm and weighting schemes

The overall system architecture of the GODoc is shown in Fig. 10. It extends PSLDoc2 (blue and green parts) with the afore-mentioned novel voting designs and weighting schemes (red part).

### Evaluation

To compare with other methods and validate the reproducibility of our experiments, we followed the evaluation measures and the dataset used in CAFA2. In this section we discuss the dataset, the cross-validation procedure for training the model, the evaluation measures, the baseline models, and the experimental design.

### Data sets

We used data from CAFA2 and CAFA3. The training data of CAFA2 includes three databases: GO Consortium, UniProt-GOA, and Swiss-Prot. As the annotation evidence codes of Swiss-Prot were more reliable than the other two, only the data from Swiss-Prot was included in our training dataset, referred to as CAFA2-Swiss. Additionally, we used the training data from CAFA3 (provides only Swiss-Prot, is referred to as CAFA3-Swiss). Regarding the test data, the benchmark dataset from CAFA2 originally was used to evaluate submitted methods, referred to as CAFA2-Benchmark. Table 3 gives a short summary of each dataset, including the number of protein sequences, the number of GO, and the median GO numbers of each protein in BPO, CCO, and MFO.

**Table 3:** Training and test dataset statistics

Dataset	Ontology	# of seqs	# of GOs	Median # of GOs
CAFA2-Swiss	BPO	40,728	15,838	25
	CCO	40,571	1892	9
	MFO	26,056	5480	8
CAFA3-Swiss	BPO	50,813	19,682	29
	CCO	49,328	2426	10
	MFO	35,086	6366	8
CAFA2-Benchmark	BPO	860	6540	29
	CCO	1259	833	11
	MFO	421	1501	8

### Five-fold cross-validation

We used five-fold cross-validation to examine the stability of the proposed method on the training dataset, which was split into five partitions. For each round, one fold was considered validation data and the other four folds were used to train the model. We repeated this for five rounds, each of which used different folds as the training and validation data. By using cross-validation to fit model parameters, we reduced the probability of model overfitting.

To avoid bias from duplicates in cross-validation, non-redundant data was generated by applying ultra-fast sequence analysis (USERACH) [18] to cluster 50% identical sequences. The size of data was reduced to around 30% (Table 4).

**Table 4:** Average protein amount of training data in cross-validation (80% of the total amount) for redundant and non-redundant datasets in different ontologies

Type	Dataset	# of redundant	# of non-redundant	Reduction ratio (%)
BPO	CAFA2-Swiss	32,582	22,231	31.77
	CAFA3-Swiss	40,650	27,158	33.19
CCO	CAFA2-Swiss	32,457	22,521	30.61
	CAFA3-Swiss	39,462	26,631	32.51
MFO	CAFA2-Swiss	20,845	14,711	29.43
	CAFA3-Swiss	28,267	19,254	31.89

### Evaluation metrics

The prediction result for each term included a confidence score between 0 and 1. Thus, a decision threshold *τ* was applied to determine the set of predicted GO terms, *P* (*τ*). Similarly, a set of experimentally determined GO terms was denoted as *T*. We focused on protein-centric evaluation, that is, an object function is calculated between *P* (*τ*) and *T* for each protein *i* and threshold *τ*. We define its precision and recall as

1 $$ {pr}_i\left(\tau \right)=\frac{\sum \limits_{v\in O}I\left(v\in {P}_i\left(\tau \right)\bullet v\in {T}_i\right)}{\sum \limits_{v\in O}I\left(v\in {P}_i\left(\tau \right)\right)} $$

2 $$ {rc}_i\left(\tau \right)=\frac{\sum \limits_{v\in O}I\left(v\in {P}_i\left(\tau \right)\bullet v\in {T}_i\right)}{\sum \limits_{v\in O}I\left(v\in {T}_i\right)} $$

where *I*(·) is an indicator function. Overall precision and recall are defined as

3 $$ pr\left(\tau \right)=\frac{1}{m\left(\tau \right)}\bullet \sum \limits_{i=1}^{m\left(\tau \right)}{pr}_i\left(\tau \right) $$

4 $$ rc\left(\tau \right)=\frac{1}{n_e}\bullet \sum \limits_{i=1}^{n_e}{rc}_i\left(\tau \right) $$

where *m*(*τ*) denotes a set of proteins with prediction confidence above threshold *τ*. As a method might predict only part of targets, an evaluation can be done under full mode (*n*_*e*_ = all dataset) or partial mode (*n*_*e*_ = *m*(0)). To provide a single evaluation metric, the maximum *F*-measure was used:

5 $$ {F}_{max}=\underset{\tau }{\max}\left\{\frac{2\bullet pr\left(\tau \right)\bullet rc\left(\tau \right)}{pr\left(\tau \right)+ rc\left(\tau \right)}\right\} $$

### Baseline models

In order to investigate the bottom line performance, we used two baseline models: Naive and BLAST. Their implementations were adopted from the Matlab code in the CAFA2 experiment [8].

- The Naive method always predicts each GO scored by its normalized frequency among the training data. As a result, query proteins are predicted with the same result.
- The BLAST method predicts GOs based on BLAST searching against the training data. We extract proteins showing local alignment identity with the query protein. The GOs of the query are predicted by assembling the GOs of similar proteins; their confidence scores are converted from the BLAST *E*-values.

### Experimental design

We conducted the following experiments to evaluate the performance of each step in the proposed framework on the CAFA2-Swiss and CAFA3-Swiss training data. The training procedure was conducted based on five-fold cross-validation (detailed in the section on Five-fold cross-validation).

- Experiment 1: Three factors of PCA reduction were evaluated, simplifying the process by using only TFPSSM 1NN: 1) the size of reduced dimensions with different explained variance ratios, 2) carried out on redundant or non-redundant training data, 3) the benefit of whitening, a preprocessing step that scales each component to the unit variance.
- Experiment 2: We sought to identify the interaction between the voting algorithm (*k* and *Q*), the weighting scheme, and propagate step for the TFPSSM vote architecture. First, *k* of Fixed-KNN (1 to 10) and the weighting scheme (*Equal*, *Inverse*, *Sqrt*) were investigated under *Max* or *Sum* propagation. After selecting the best weighting scheme, *Q* of Dynamic KNN (Q1, Q2, Q3) was investigated in the same way. The benefit of incorporating protein domain information was judged by a comparison between *FunOverlap* and the selected weighting scheme. Last, the performance of Hybird-KNN was evaluated.

Finally, a model was trained according to the best setting learned from the previous experiments. Then, it was evaluated on the CAFA2-benchmark so it could be compared with other methods reported in CAFA2 on the same basis.

## Supplementary information

**Additional file 1: Table S1.** The performance of the submitted Model 1 based on minimum normalized semantic distance metric adapted from CAFA3 Results Release.

## Data Availability

The GODoc method is available at https://github.com/changlabtw/GODoc.

## Abbreviations

CAFA: Critical Assessment of Functional Annotation
GO: Gene Ontology
GODoc: Gene Ontology prediction based on Document classification method
FunFam: Functional Family
KNN: K-nearest-neighbor
